# Organizing Humanitarian Cochlear Implant Missions: “Green Cochlea” Guide

**DOI:** 10.5334/aogh.4925

**Published:** 2025-10-03

**Authors:** Isra Aljazeeri, Yassin Abdelsamad, Eman Hajr, Mohammad Alzahrani, Tawfiq Khurayzi, Ahmad M. Aldhafeeri, Rayan Alhussaini, Musaed Alzharani, Fida Almuhwas, Farid Alzhrani, Abdulrahman Hagr

**Affiliations:** 1 Department of Otolaryngology – Head and Neck Surgery, College of Medicine, King Saud University, Riyadh, Saudi Arabia; 2 King Abdullah Ear Specialist Center (KAESC), King Saud University Medical City, Riyadh, Saudi Arabia; 3 Aljaber Ophthalmology and Otolaryngology Specialized Hospital, Ahsa, Ministry of Health, Saudi Arabia; 4 Research Department, MED-EL GmbH, Riyadh, Saudi Arabia; 5 Otolaryngology Department, College of Medicine, Imam Mohammad Ibn Saud Islamic University, Riyadh, Saudi Arabia; 6 College of Medicine, King Saud University, Riyadh, Saudi Arabia; 7 Otolaryngology, Neurotology & Lateral Skull Base Surgery, King Fahad Central Hospital, Jizan, Saudi Arabia; 8 Hafr Albaten Central Hospital, Ministry of Health, Hafr Albaten, Saudi Arabia; 9 Cochlear Implant Center, National Guard Hospital, Madinah, Saudi Arabia; 10 Department of Otolaryngology – Head and Neck Surgery, King Fahad Specialist Hospital, Dammam, Saudi Arabia

**Keywords:** hearing loss, cochlear implant, humanitarian, healthcare, guide, sustainability, green cochlea

## Abstract

*Objective:* This paper aims to provide a step-by-step guide for organizing humanitarian cochlear implant missions (HCIMs).

*Methods:* A panel of experienced professionals collaborated through informal, focused group discussions to create this comprehensive guide for organizing HCIMs. It includes a flowchart, a checklist, and detailed descriptions of each step, offering a clear and structured framework for the process. Subsequently, additional advisors were invited to evaluate this outline and provide their input.

*Results:* All participants approved the final version after the outline was improved. This guide proposes the following eight steps: (1) aim and funding, (2) project study, (3) preparation, (4) local system setup (local team recruitment and patient recruitment), (5) pilot campaign, (6) full-scale campaign, (7) On-site process, and (8) follow-up. Thirteen items are specified across the eight main steps in a detailed checklist. All participants approved the final version after the outline was improved.

*Conclusion:* The proposed good practice guide (GPG) includes a flowchart and a checklist that provides a comprehensive manual for establishing, conducting, and organizing international HCIMs. Understanding the process that is expected to be followed when planning an international HCIMs enables the involved parties to organize and assign tasks and create a schedule that allows them to finish their tasks on time and with the best quality. This guide could be a basis to describe an organized process, resulting in professional and distinguished campaigns.

## Introduction

According to reports from the World Health Organization, many people have hearing loss (HL), which impacts their quality of life in various ways. This challenge becomes even more profound for those living in environments marked by harsh conditions and instability, increasing their struggles and making their circumstances more difficult. Consequently, there is an urgent need for humanitarian efforts to alleviate these hardships, restore what can be repaired, and offer these individuals a chance and hope for a better present and future.

Humanitarian medical missions need different settings than local permanent medical care providers, and that in many perspectives, such as logistics, healthcare structure, and time and resource availability [1]. Unfortunately, in developing countries, access to healthcare is often hindered by a lack of facilities, insufficient equipment, unaffordable costs, and a lack of awareness about available treatments. These factors significantly limit the implementation of humanitarian medical missions in general, and the challenges become even more pronounced when addressing specialized services such as cochlear implant services.

Cochlear implants (CIs) are the most successful neural prostheses available [2]. CI devices enable hearing by sending sounds as electrical signals directly to the cochlear nerve endings [3]. Worldwide, it is estimated that fewer than 5% of those eligible for CI have been implanted, with the majority of the implanted patients being from high-income countries. This demonstrates the extent of the demand for humanitarian CIs [4].

A key component of the treatment, and a complex challenge in HCIMs, is the need for extensive preoperative evaluation. On the other hand, continued postoperative services such as mapping of the devices, rehabilitation programs, and auditory training should be provided [5].

Given the substantial lack of information and resources in the current literature regarding how to properly conduct HCIMs, the primary objective of this study is to bridge that gap by offering a thorough and detailed roadmap for successfully carrying out these initiatives. This includes providing comprehensive, step-by-step guidance to address the unique challenges and complexities associated with these programs, ensuring that they are effectively planned, implemented, and sustained. By doing so, this study seeks to serve as a resource and reference for professionals, organizations, and stakeholders involved in delivering critical medical intervention to underserved populations.

## Methodology

A qualitative study design was adopted to develop this guide, undertaking focused group discussions consisting of the organizers and participants in the largest humanitarian CI mission, which was performed by the King Salman Humanitarian Aid and Relief Centre, Kingdome of Saudi Arabia (KSA), in Turkey for the Syrian people in 2024. Purposeful variation sampling was used to recruit the sample across all levels of the organization. Attention was given to include those in the lower grades of the organization who participated in applying the recommendations included in this good practice guide (GPG) to get feedback regarding the practicality, benefits, and difficulties encountered while applying the Green Cochlea GPG (GCGPG). The focused group included the chair and co-chair of the organizing committee (Dr. F.A and Prof. F.A), the committee advisor (Prof. A.H), two participating otologists (Dr. I.A. and Dr. A.A.), and a participating coordinator (Mr. M.A.). The focus group approach involved asking the participants to summarize the organizational process and to indicate the benefits, importance, and limitations in conducting each course of action. This feedback was analyzed to explore the steps of the organizational process and formulate the GPG. After drafting the GPG, three additional advisors with experience in the organizational process were involved to provide their feedback (Dr. E.H., Dr. T.K., Dr. R.A., Dr. M.A, and Dr. Y.A.). All the participants then approved the final version of the GCGPG.

## Result

**Figure 1:**
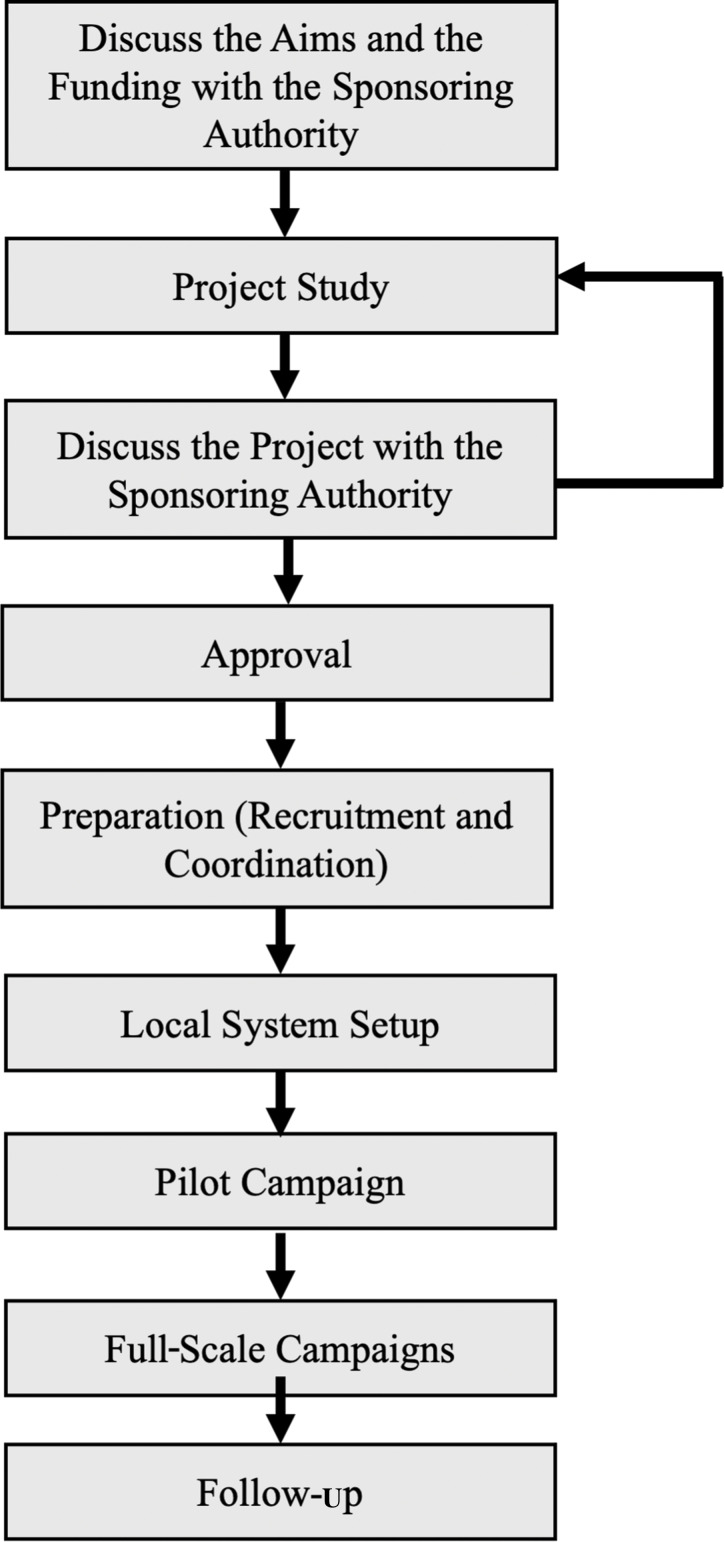
The flowchart for organizing Green Cochlea Good Practice Guide (GCGPG).

The open discussions of the focused group resulted in structuring the organizational process into eight steps, which are listed below and summarized in Figure 1.

### 1. Aim and funding

The sponsoring authority hosting the mission provides the vision of the mission and its target. The mission needs to be evaluated from geopolitical considerations to ensure that the good intentions behind it are well presented.

The budget is the main determining factor for the scale of any humanitarian mission. The sponsoring authority needs to know the main costs and allow for flexibility in unexpected expenses. The predictable costs can vary according to the available facilities in the targeted area.

The costs of a cochlear implantation mission may include, but are not limited to, the following:

- The hospital service costs include admission, preoperative lab tests, imaging, surgical room and requirement costs, and postoperative inpatient and discharge medications.
- The cost of the CI device, including its accessories and technical support services.
- The expenses associated with providing the surgical equipment, whether through permanent acquisition or temporary transfer of the necessary tools.
- The salary of the medical team (if the participants are contributing as humanitarian nonprofit participants, this might not be needed).
- The cost of organizing, transporting, and accommodating the medical team.
- The cost of the patients’ and their families’ transport and accommodation.
- The cost of long-term aural rehabilitation care.

Additional items might include the surgeon’s preferential instruments, which are not mandatory, such as suction-retractor, light source, surgical loupes, as well as Hagr’s retractor forceps and retractor with its portable light source (Table 1).

**Table 1:** The list of equipment needed, which can be modified according to the participating surgeons’ preferences.

ITEM	SPECIFICATIONS
Anesthesia machine	Per each surgical room
Anesthesia equipment: airway (oral, nasal), endotracheal tube, stylet, face mask, pulse oximeter …	Per each patient
Surgical drapes, microscope drapes, surgical gowns, and gloves	Per each patient
Otological surgical microscope	Per each surgical room
Mastoidectomy surgical set (including surgical blade handle, toothed and non-toothed forceps, two cat’s paw retractors, two self-retaining retractors, periosteal elevator or freer elevator, macro- and micro-suctions, suture holder)	Reusable with sterilization
Electrode insertion set (including insertion forceps and claw).	Reusable with sterilization
Surgical otologic drill and its burs (minimum: size 7 or 6 cutting, 4, 2, 1.5 diamond)	Per each surgical room. The drill itself is reusable with sterilization, while the burs can be either reusable or disposable according to the brand
Facial nerve monitor (including its probes and electrodes)	The machine per each surgical room; the probes and electrodes per each patient
Monopolar diathermy and/or blades	The diathermy machine per each surgical room; the diathermy blade per each patient
Sutures and absorbable sponge	Per each patient
Camera cover or a sterile glove to cover the sound processor used for intraoperative monitoring	Per each patient
Bone wax and hemostatic agents	Only when needed in rare cases with difficult hemostasis

Using monopolar cautery alone can decrease the operative time by saving the time needed for preparing and injecting local anesthesia and by providing a bloodless field. This practice depends on surgeons’ preference and experience.

While the majority of costs can be estimated in advance, it is essential to add around a 20% extra margin to allow for some flexibility to accommodate unexpected expenses that may arise during the pilot campaign. This margin can be adjusted based on the experience with the pilot campaign for the rest of the mission. Unforeseen costs can occur at various stages, such as the need to repeat imaging, conduct additional pre- or postoperative tests, perform revision surgeries, manage complications, or replace broken or malfunctioning equipment. Being prepared for these possibilities is crucial to ensuring the smooth execution of the mission.

### 2. Project study

In this step, the organizing team must develop a comprehensive outline of the process to identify the necessary arrangements, determine which organizations will be involved, estimate the overall mission costs, and establish the enrollment criteria for candidates.

This process typically begins with designing a strategy for detecting patients who meet the eligibility criteria. Patient detection is a time-intensive process; therefore, collaboration with multiple clinics might be needed to ensure adequate outreach. For HCIMs that operate as temporary services, partnering with local healthcare providers is essential to identify and refer potential candidates efficiently. CI candidate detection becomes significantly more efficient if the target population already benefits from a neonatal hearing screening program and has access to diagnostic audiological evaluations, as these can facilitate the early detection of suitable candidates for the program. In situations where local hearing evaluation services are unavailable, establishing a semi-permanent hearing assessment clinic becomes essential to identify and detect CI candidates effectively. To address the broader community needs, it is also advisable to implement a parallel humanitarian hearing aid distribution program. This program would provide hearing aids to individuals identified as requiring them during the detection process for CI candidates, ensuring that more individuals benefit from improved hearing health services.

### 3. Preparation

Once the project study has been analyzed and the patient detection strategy approved, preparations for launching the campaign should focus on four key aspects:

- Setting a tentative timeline for the campaign
- Recruiting the medical team
- Collaborative multidisciplinary approach for patient evaluation (CI committee)
- Additional preparations, including logistical and technical arrangements

#### A. Setting a tentative timeline for the campaign

The organizers need to establish a potential outline to ensure smooth coordination and implementation of activities and to allow adequate time to prepare human power and all required resources.

#### B. Recruiting the medical team

The medical team for an HCIM includes various care providers, including surgeons, surgical nurses, anesthesiologists, anesthesia technicians, audiologists, speech-language therapists, and coordinators. The organizers should assemble a qualified team with all the necessary professionals to provide the required services. Evaluating the local hosting facility will help identify any medical team members who are lacking.

CI surgery is considered a highly specialized procedure, requiring meticulous attention, particularly when selecting candidates who may have been waiting for such an opportunity for an extended period. For many, this could be their only chance; therefore, assembling a highly qualified and experienced surgical team is of paramount importance. Surgeons involved in the HCIM must hold a high-ranked otology fellowship certification, be fully accredited consultants, and have extensive independent practice experience. This includes handling a diverse range of cases, such as those involving malformations, enabling them to address complex surgical challenges, navigate anatomical anomalies, and achieve low complication rates. The participating surgeons also need to have leadership qualities to enable them to help in leading their team. This includes good rapport and communication skills.

Having a national surgeons’ registry that shows each surgeon’s surgical load and complication rates would help in determining the appropriate participants in the HCIMs. The latest five years of the surgeons’ practice should be particularly studied. If no national registry is available, some data can be retrieved from the CI manufacturers, upon surgeons’ approval, including the number of cases, the rates of reoperation, and the number of improper placements (extracochlear placement, tip fold-over, and incomplete insertion). It should be noted that the presence of predisposing causes, such as cochlear anomalies, intracochlear fibrosis, or cochlear ossification, should be accounted for, and these are justifiable causes for improper placements and do not reflect decreased surgical skills. Additionally, the number of intraoperative openings of back-up devices for each surgeon should also be evaluated, which may reflect decision-making deficiencies.

In addition to the critical role of an experienced surgeon, ensuring the smooth execution of the mission requires assembling a comprehensive team of care providers proficient in handling similar surgical procedures as part of their routine practice. This multidisciplinary team is essential for providing seamless coordination and high-quality care throughout the process. It is particularly important to include operating room nursing staff skilled in supporting complex surgical interventions, audiologists with expertise in pre- and postsurgical auditory assessments, and speech-language pathologists who can contribute to the rehabilitation process and help optimize patient outcomes. Their collective familiarity with these types of procedures ensures efficiency, minimizes potential challenges, and enhances the overall success of the mission.

#### C. Collaborative multidisciplinary approach for patient evaluation (cochlear implant committee)

To ensure optimal outcomes, a dedicated team comprising a surgeon, an audiologist, and a speech-language pathologist must be established to collaboratively review and discuss each individual patient’s case. This process can be conducted in two phases to ensure thorough preparation and readiness of every patient before the actual campaign.

**Phase 1: Initial Evaluation and Screening**

The first phase involves close collaboration with the local team to complete all necessary preoperative preparations. This includes capturing comprehensive preoperative work-up data and designing a standardized data collection form. An online meeting is held with a subgroup of contributors, including the surgeon, audiologist, and speech-language pathologist. During this meeting, patient charts are screened to categorize each case into one of three outcomes:

1. Full preliminary acceptance for surgery
2. Conditional acceptance, pending on-site reevaluation during the campaign
3. Rejection, based on specific contraindications

**Phase 2: On-Site Evaluation with “One-Stop Clinic” Strategy**

The second evaluation phase occurs during the campaign, using a streamlined “one-stop clinic” approach. In this setup, the patient and their legal guardian meet with the three-member committee in a single session to minimize time between evaluations.

1. **Audiological Assessment**
  - The audiologist evaluates the patient, ensuring the accuracy and reliability of all preoperative auditory tests.
2. **Speech-Language Evaluation**
  - The speech-language pathologist conducts an in-person assessment of the patient’s communication and speech abilities.
3. **Surgical Assessment**
  - The surgeon confirms the patient’s medical history, ensures proper vaccinations, performs a physical examination, reviews imaging findings, and counsels the family regarding the surgical aspects of cochlear implantation.
  - A demonstration of the surgical process using a dummy device can help families understand the procedure.

If all evaluations are satisfactory and the family fully understands the surgical process, consent for surgery is obtained at this stage.

#### D. Additional preparations: logistical and technical arrangements

- **Use of Telemedicine**
  To ensure continuity of care, telemedicine can bridge the gap by enabling remote consultations and follow-ups. As part of the support plan, it is recommended to establish a location within the patients’ local area with reliable internet connectivity, ensuring they have access to telemedicine services when needed.
- **Supporting Team from Cochlear Implant Manufacturers**
  Successful CI missions require substantial support from CI manufacturers. Their support includes providing technical expertise, assisting with device functionality, objective measurements, troubleshooting, and supplying necessary consumables and accessories. Close collaboration with these companies is essential to address any technical challenges and ensure smooth execution of the mission.

### 4. Local system setup (local team recruitment and recruiting the patients)

An essential prerequisite for the success and sustainability of any HCIM is the partnership and engagement with a powerful local sponsor. This sponsor needs to have political authority and influence. It can be a governmental body, a ministry of health official, or a high-level local medical leader. Their role is indispensable in navigating administrative hurdles, securing necessary permits, facilitating customs clearance for equipment, and ensuring that the mission aligns with national health priorities. This sponsor acts as the local project’s anchor, providing legitimacy within the local context and facilitating the progress of the mission through inevitable logistical and political challenges.

When humanitarian missions are applied in foreign countries, there is also a need for coordination with the local healthcare system. CI service is particularly in need of local support due to its requirement for long-term follow-up and postoperative services.

The local team needs to have official local authority and act within the governmental system of the targeted country. Their task starts with arranging patient detection and ensuring all the required preoperative evaluations are available. In the next step, the local team would arrange for patients’ transportation to the hosting care facility and their housing. They would also help in supporting long-term care and connecting the patients to the needed services when needed. Knowing the language and culture of the targeted population enables the local team to communicate effectively, provide better counselling, build a high level of trust, and avoid any misunderstandings.

### 5. Pilot campaign

When a large-scale mission is planned, a pilot subproject with a small number of surgeries will help reveal the unexpected challenges and costs. This will help refine the plan for the full-scale campaign. A large-scale mission can also be divided into multiple rounds of smaller campaigns. The pilot campaign can be preceded by a visit from one expert who can evaluate all the needs and address all the organizing team’s concerns and questions.

### 6. Full-Scale campaign

Following all the previous steps will ease the execution of the full-scale campaign. However, the project will always need to be evaluated continuously to detect shortcomings or areas for improvement.

The safety and well-being of the humanitarian project participants should be a priority. The caregiving team should have proper protection against natural disasters and unsafe geopolitical environment. The participants should also receive the necessary vaccinations and have access to appropriate medical care when needed

### 7. On-Site process

The humanitarian CI mission involves various care providers. Coordinating the recruitment and transportation of this team to the mission’s local area presents significant challenges. Each of these highly specialized professionals has their own commitments and responsibilities. The costs associated with transferring patients and arranging accommodations can also be substantial. Therefore, executing the on-site process within the shortest timeframe is essential.

**Figure 2:**
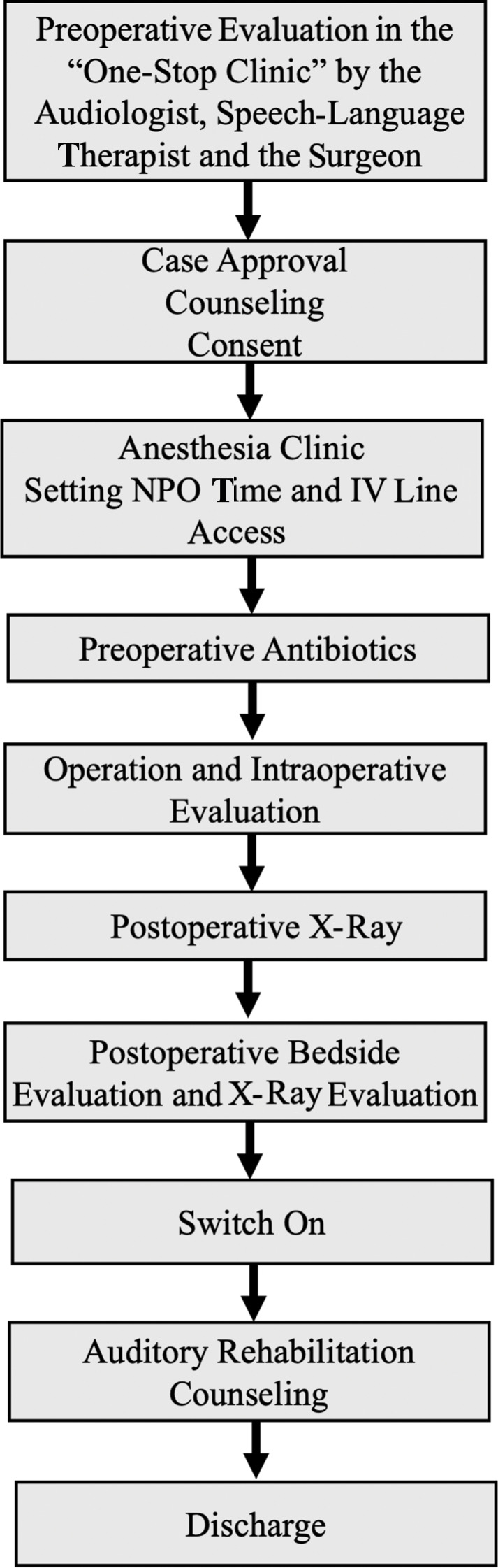
Summarizes the case journey from admission to the discharge.

Numerous tasks need to be performed for each patient, starting from the preoperative candidacy evaluation to discharge (Figure 2). The case turnover can be overwhelming when large-scale projects are planned within a limited time. For this reason, a live online tracking system is essential to keep all team members updated.

### 8. Follow-Up

One of the peculiar aspects of CI service is the need for long-term follow-ups. This continual care is a cornerstone of the eventual outcome of the CI surgery. For this reason, it is of utmost importance to plan this step of care from the beginning.

Establishing a permanent local supportive team of audiology, speech-language therapy, and a manufacturers’ support branch is the ultimate goal for providing the best care possible. In cases when this is not possible, both auditory rehabilitation sessions and programming can be done through telemedicine.

***The Green Cochlea Concept***

The concept of Green Cochlea was developed to include a collection of practices aimed at the optimization of cochlear implantation.

This concept includes the following:

- Sustainability of the Service
  Streamline processes to reduce redundancies and avoid logistical mishaps. Establishing efficient protocols ensures that missions operate smoothly, thus enhancing credibility and trust within the community.
  Prioritize local partnerships to foster a sense of ownership and continuity within the communities served. Involve local healthcare providers, as they play a crucial role in follow-up care and support for patients after surgery.
  Encourage training for local teams to ensure they can sustain CI services independently, thus empowering them.
  Additionally, there is a need to avoid troubles and mishaps that may prevent future missions. One important aspect of sustainability is leaving dignified impressions and maintaining a positive attitude with good personal connections.
- Minimalism
  Minimalism refers to the surgical practices designed to achieve maximum outcomes through minimal interventions.
  This includes using the smallest skin incision, approximately 3 cm, and ensuring secure placement of the internal receiver stimulator (IRS). Such an approach aims to reduce the risk of wound and flap complications, including hematomas, seromas, surgical site infections, IRS displacement, and flap necrosis.
  Another proposed practice is to forgo dressing; with minimal skin incisions and the IRS housed in a tight periosteal pocket, the likelihood of hematomas and seromas becomes lower. This precludes the need for postoperative dressing.
  Additionally, expediting the surgical process minimizes anesthesia time and operative bleeding. The speed at which the surgery is performed should maintain the utmost importance of safety and avoid omitting any steps. Key factors in achieving swift surgeries include recruiting skilled and efficient surgeons, utilizing high-speed drills, performing cortical mastoidectomies with the naked eye, adhering strictly to surgical landmarks, and assigning complex cases to the most skillful surgeons.
- Early Activation: This practice significantly reduces the duration of the mission, contributing to the feasibility and sustainability of care for both care providers and recipients.

## Discussion

HL is disproportionately distributed across the world, with low- and middle-income nations accounting for roughly 60% of people with bilateral moderate-to-profound HL [6, 7]. Disabling HL in adults and children is substantially higher in low-income regions, with rates more than twice those reported in high-income countries. [WHO8] [8, 9]. The prevalence of debilitating HL decreases significantly as gross national income (GNI) increases in adults and children. Furthermore, as parental literacy rates rise, the incidence of debilitating HL in children decreases [WHO8].

This disparity highlights the need for humanitarian initiatives focused on increasing access to CIs in underserved regions, where the prevalence of hearing impairment remains alarmingly high [4, 10].

When planning humanitarian missions, great care must be taken to avoid harming the vulnerable population [11]. The organizers need to take into consideration the “Seven Sins of Humanitarian Medicine,” demonstrated by Welling et al., including leaving a mess behind; failing to match technology to local needs and abilities; failing to have a follow-up plan; allowing politics, training, or other distracting goals to trump service; going where we are not wanted or needed; and doing the right thing for the wrong reason [12].

For the success of hearing-related humanitarian missions, it is imperative to have extremely long-term follow-up, which is not possible except with the establishment of appropriate local auditory services [10, 13]. Furthermore, repeated visits to the same location increase the efficiency of the mission by identifying drawbacks, refining the process, and increasing familiarity within the local area with the intended care [14, 15].

In this study, we aimed to describe in detail the process of organizing HCIMs. This guide delineates this process in eight structured steps and provides a flowchart to facilitate the planning and execution of any HCIM. These steps summarize the collective experiences of the authors, who have been the organizers of the largest HCIM, highlighting the critical elements that contributed to the success of these initiatives.

Moreover, the necessity for collaborative efforts among multidisciplinary teams cannot be overstated. As demonstrated in our guide, the engagement of various healthcare professionals, including surgeons, audiologists, speech-language pathologists, and local healthcare providers, forms the backbone of an effective HCIM. This synergy is vital for addressing the multifaceted needs of the patient population and ensuring comprehensive care throughout the surgical and rehabilitation processes.

## Conclusion

HCIMs are highly complex professional endeavors that need to be planned meticulously. This GPG provides a comprehensive framework for organizing HCIMs, emphasizing a methodical approach that includes essential steps from funding to follow-up. This guide has been developed using focused group discussions with an expert team who participated in the largest humanitarian CI mission. This guide aims to empower stakeholders, facilitating the successful implementation of campaigns that significantly improve access to CI services in humanitarian settings.

## Statements and Declarations

- The authors have no competing interests to declare.
- The authors have no relevant financial or nonfinancial interests to disclose.
- The authors did not receive support from any organization for the submitted work.
- The data for this article are available upon request from the corresponding author.

## Highest Degree of the Authors

Isra Aljazeeri MD

Yassin Abdelsamad PhD

Eman Hajr MD

Mohammad Alzahrani MD

Tawfiq Khurayzi MD

Ahmad M. Aldhafeeri MD

Rayan Alhussaini MD

Musaed Alzharani MD

Fida Almuhwas MD

Prof. Farid Alzhrani MD

Prof. Abdulrahman Hagr MD
